# IDH1 R132H predicts sensitivity to Bcl-xL inhibition-mediated programmed cell death

**DOI:** 10.18632/oncotarget.23399

**Published:** 2017-12-18

**Authors:** Georg Karpel-Massler, Chiaki Tsuge Ishida, Markus D. Siegelin

**Affiliations:** Markus D. Siegelin: Department of Pathology & Cell Biology, Columbia University Medical Center, New York, NY, USA

**Keywords:** glioblastoma, astrocytoma, IDH1, Bcl-2, Bcl-xL

The anti-apoptotic Bcl-2 family of proteins, such as Bcl-2, Bcl-xL and Mcl-1, are pivotal in cell death regulation in cancer. More than three decades ago, the first of these cell death regulators was identified as part of a translocation, involving chromosome 14 and 18 [1]. This genetic alteration drives the overexpression of Bcl-2 and is part of the diagnostic panel for a particular Non-Hodgkin lymphoma, follicular lymphoma, which is histologically characterized by neoplastic follicles. While many oncogenes mainly drive cell cycle progression and hence cellular proliferation, the anti-apoptotic Bcl-2 family protein is different since it mainly antagonizes cell death downstream at the level of the outer membrane permeabilization of mitochondria (MOMP) by sequestering the pro-apoptotic Bcl-2 family members Bax and Bak, which together drive MOMP and subsequent release of cytochrome-c into the cytosol from the inner mitochondrial membrane space to engage in intrinsic apoptosis by activation of initiator- and effector caspases [2]. After the identification of Bcl-2, other anti-apoptotic members were discovered, such as Bcl-xL and Mcl-1. While Bcl-xL shares many similarities to Bcl-2, particularly with regards to BAX/BAK sequestration, Mcl-1 is different in that it binds preferentially to BAK [2, 3]. Another molecule that interacts with Mcl-1 is Noxa, which has been shown to counteract the Mcl-1-Bak interaction [4]. All these proteins are up regulated to various degrees in cancer and therefore offer a targetable therapeutic window. While Bcl-2 appears to be more important in hematological malignancies, Bcl-xL is more critical in solid tumors. Dual inhibition of Bcl-2/Bcl-xL results in a compensatory inhibition of cell death mediated by Mcl-1. Therefore, to accomplish most efficient tumor cell killing, ideally all three members have to be inhibited. About a decade ago, BH3-mimetics were first described, such as ABT-737 and the oral derivative ABT-263. They inhibit Bcl-2 and Bcl-xL, but not Mcl-1, which is the main mediator of therapeutic resistance to these compounds [5, 6]. These class of molecules are considered to be a significant advance in drug discovery since they are capable of binding to their target in the low nanomolar range. Cells being susceptible respond with rapid apoptosis upon treatment.

Recently published work by our group has now unraveled a selective vulnerability of IDH1 mutant glioma/glioblastoma model systems [7]. The IDH1 mutation is particularly common in secondary glioblastomas and low-grade gliomas, such as oligodendrogliomas and the IDH1 R132H mutation can be readily detected by immunohistochemistry on patient samples. Moreover, tumors, harboring the IDH1 mutation, display an accumulation of an oncometabolite, 2-R-2-hydroxyglutarate (2-HG), which can reach levels in the millimolar range. Concerning the prognosis of glioma patients, presence of the IDH1 mutation was shown to be related to a significantly prolonged survival. The present literature suggests that many features of IDH mutant gliomas are in fact mediated by 2-HG, e.g. the generation of a hypermethylated phenotype, CIMP.

We have found that IDH1 mutated astrocytoma specimens display lower protein levels of Mcl-1 as compared to IDH1 wild-type samples. Similarly, genetically engineered cell lines of mutated IDH1 R132H showed lower levels of Mcl-1. Utilizing chemically synthesized 2-HG that has been engineered to penetrate the cell membrane, we found that physiologically relevant concentrations of 2-HG (at levels that are encountered in tumors) down-regulated Mcl-1 protein levels as well. Consequently, both 2-HG and IDH1 R132H enhanced apoptosis by Bcl-xL inhibition as well as by the BH3-mimetic, ABT263, in multiple glioma model systems. Interestingly, neither IDH1 R132H nor 2-HG sensitized for Bcl-2 inhibition mediated by the selective BH3-mimetic, ABT199, suggesting that Bcl-xL indeed mediates the synthetic lethal interaction in the context of glioma. Our results should be seen in context of another recently published paper that has observed that Bcl-2 and Bcl-w inhibition is synthetically lethal in IDH-mutant model systems of acute myeloid leukemia, which was derived from a genome-wide lentiviral shRNA library screen [8]. Akin to gliomas, AML often harbor the IDH1 mutation as well.

**Figure 1:**
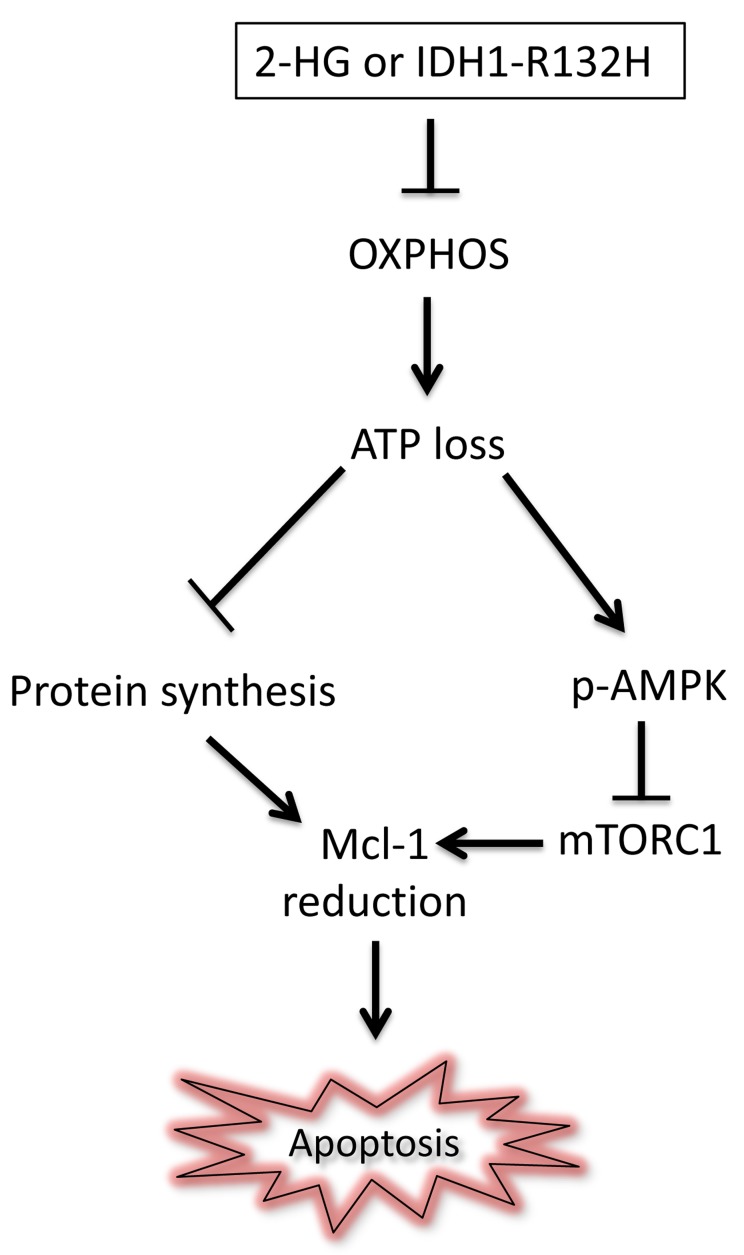
The IDH1 mutation related metabolite 2-HG primes tumor cells to Bcl-xL inhibition by depleting them of energy: Apoptosis in cells is tightly regulated and Mcl-1 inhibits cancer cell death (apoptosis) The presence of mutant IDH1 and its associated oncometabolite 2-HG causes an inhibition of oxidative phosphorylation (OXPHOS) with a subsequent decline in ATP (adenosine triphosphate) levels in cancer cells. In turn, this leads to suppression of protein synthesis and blunting of mTOR signaling via activation of the AMPK kinase (phosphorylation at threonine 172), culminating in a significant decline in Mcl-1. In turn, Mcl-1 down-regulation sensitizes glioblastoma cells to apoptosis induction by Bcl-xL inhibition through the BH3-mimetic, ABT263.

Our results show that IDH1 R132H and 2-HG interfere with tumor cell metabolism and demonstrate that IDH1 R132H and 2-HG suppress energy production via oxidative phosphorylation, which subsequently leads to a decline in energy levels (Figure 1). However, the precise underlying mechanism as to how 2-HG regulates oxidative phosphorylation remains to be determined. Some evidence suggests that it may involve a direct enzymatic inhibition of respiratory complexes. However, it is conceivable that Mcl-1 itself might have an impact on cellular respiration as well since some pools of Mcl-1 localize to the mitochondrial matrix. Another hypothesis is that the hypermethylated phenotype by IDH1 mutated gliomas contributes to these metabolic alterations. Low energy levels are often accompanied by a reduction of protein synthesis as this process requires energy delivering phosphoanhydride bonds in the form of tri-phospho nucleotides, such as GTP. Supporting this general notion, both 2-HG and IDH1 R132 lowered protein synthesis and hence the expression of proteins that have a short half-life, such as Mcl-1. In turn low levels of Mcl-1 prime tumor cells to apoptosis induction by Bcl-xL inhibition.

The appeal of this outlined approach is the potential selectivity for tumor cells and the fact that in the context of the recently revised WHO classification for central nervous system tumors IDH mutations are part of the standard diagnostic procedure for gliomas, thus ensuring that almost all mutations for IDH1 will be detected in the course of establishing a diagnosis. In summary, our work provides a foundation and justification for testing Bcl-xL inhibition in patients with IDH1 mutated gliomas.
